# An Internet-supported Physical Activity Intervention Delivered in Secondary Schools Located in Low Socio-economic Status Communities: Study Protocol for the Activity and Motivation in Physical Education (AMPED) Cluster Randomized Controlled Trial 

**DOI:** 10.1186/s12889-015-2583-7

**Published:** 2016-01-06

**Authors:** Chris Lonsdale, Aidan Lester, Katherine B. Owen, Rhiannon L. White, Ian Moyes, Louisa Peralta, Morwenna Kirwan, Anthony Maeder, Andrew Bennie, Freya MacMillan, Gregory S. Kolt, Nikos Ntoumanis, Jennifer M. Gore, Ester Cerin, Thierno M.O. Diallo, Dylan P. Cliff, David R. Lubans

**Affiliations:** 1 Institute for Positive Psychology and Education, Australian Catholic University, 25A Barker Road, Strathfield, NSW 2135 Australia; 2 Faculty of Education and Social Work, University of Sydney, Sydney, NSW 2006 Australia; 3 School of Science and Health, Western Sydney University, Locked Bag 1797, Penrith, NSW 2751 Australia; 4 School Computing, Engineering and Mathematics, Western Sydney University, Locked Bag 1797, Penrith, NSW 2751 Australia; 5 School of Psychology and Speech Pathology, Curtin University, Kent Street, Bentley, Perth, Western Australia 6102 Australia; 6 School of Education, University of Newcastle, Callaghan, NSW 2308 Australia; 7 Center for Physical Activity and Nutrition, Faculty of Health, Deakin University, 221 Burwood Highway, Burwood, Victoria 3125 Australia; 8 School of Public Health, The University of Hong Kong, Pokfulam Road, Hong Kong, China; 9 Early Start Research Institute, School of Education, University of Wollongong, Wollongong, NSW 2522 Australia; 10 Priority Research Center in Physical Activity and Nutrition, School of Education, University of Newcastle, Callaghan, NSW 2308 Australia

**Keywords:** Physical activity, Motivation, Teacher professional development, Web 2.0, Online, Internet, Mobile application, App

## Abstract

**Background:**

School-based physical education is an important public health initiative as it has the potential to provide students with regular opportunities to participate in moderate-to-vigorous physical activity (MVPA). Unfortunately, in many physical education lessons students do not engage in sufficient MVPA to achieve health benefits. In this trial we will test the efficacy of a teacher professional development intervention, delivered partially via the Internet, on secondary school students’ MVPA during physical education lessons. Teaching strategies covered in this training are designed to (i) maximize opportunities for students to be physically active during lessons and (ii) enhance students’ autonomous motivation towards physical activity.

**Method:**

A two-arm cluster randomized controlled trial with allocation at the school level (intervention vs. usual care control). Teachers and Year 8 students in government-funded secondary schools in low socio-economic areas of the Western Sydney region of Australia will be eligible to participate. During the main portion of the intervention (6 months), teachers will participate in two workshops and complete two implementation tasks at their school. Implementation tasks will involve video-based self-reflection via the project’s Web 2.0 platform and an individualized feedback meeting with a project mentor. Each intervention school will also complete two group peer-mentoring sessions at their school (one per term) in which they will discuss implementation with members of their school physical education staff. In the booster period (3 months), teachers will complete a half-day workshop at their school, plus one online implementation task, and a group mentoring session at their school. Throughout the entire intervention period (main intervention plus booster period), teachers will have access to online resources. Data collection will include baseline, post-intervention (7–8 months after baseline) and maintenance phase (14–15 months after baseline) assessments. Research assistants blinded to group allocation will collect all data. The primary outcome will be the proportion of physical education lesson time that students spend in MVPA. Secondary outcomes will include leisure-time physical activity, subjective well-being, and motivation towards physical activity.

**Discussion:**

The provision of an online training platform for teachers could help facilitate more widespread dissemination of evidence-based interventions compared with programs that rely exclusively on face-to-face training.

**Trial registration:**

Australia and New Zealand Clinical Trials Registry-ACTRN12614000184673. Registration date: February 19, 2014.

**Electronic supplementary material:**

The online version of this article (doi:10.1186/s12889-015-2583-7) contains supplementary material, which is available to authorized users.

## Background

### Purpose

Schools represent an ideal environment for physical activity promotion among youth. They provide access to the majority of the population and have the facilities, trained personnel and curriculum to address public health objectives [1]. Physical education (PE) is the primary vehicle responsible for physical activity promotion in schools and has the potential to provide students with regular opportunities to be physically active. Unfortunately, in many of these lessons students do not engage in sufficient moderate-to-vigorous physical activity (MVPA) to achieve health benefits [2–4]. In this trial we will test the effect of a PE teacher professional development intervention, delivered partially via the Internet, on students’ MVPA during PE lessons. Known as the Activity and Motivation in Physical Education (AMPED) Project, this intervention is also designed to enhance students’ motivation to be physically active during PE lessons and their leisure-time (i.e., outside school hours). If MVPA during PE and leisure time can be increased, youth can be expected to realize a variety of benefits, including enhanced self-concept [5], better quality of life [6], greater engagement at school [7], better academic results [8] and improved physical health [9].

### Overview of previous research

#### Focusing on lesson structure to improve PE lessons provides opportunities for MVPA

Numerous studies have assessed the effect of interventions designed to increase MVPA during PE lessons [3]. These investigations have included large trials in the United States focused on improving PE teachers’ lesson planning and delivery [10, 11]. For example, the Middle School Physical Activity and Nutrition intervention [10] created teacher awareness, helped teachers to design and implement active PE lessons, and developed teachers’ class management skills. Previous interventions focusing on lesson planning and delivery have successfully increased the proportion of time students spent in MVPA during PE lessons. Indeed, a recent meta-analysis [3] found a pooled effect of *d* = 0.6 when comparing the physical activity levels of intervention versus control students. This difference was equivalent to approximately 24 % more MVPA during PE lessons.

#### Student motivation towards PE is associated with MVPA during lessons

Epidemiological evidence suggests that the largest decrease in youth physical activity occurs during early adolescence [12]. This trend parallels age-related declines in adaptive motivation for school and PE, in particular [13]. Few interventions have been specifically designed to motivate students to take advantage of opportunities for MVPA during PE lessons [14]. This omission ignores the evidence that student motivation is an important correlate and likely determinant of MVPA during PE [15]. For example, Lonsdale and colleagues [16] showed that students motivated by autonomous factors (e.g., intrinsic motivation) were 20 % more active than students in the same lessons who were motivated by external factors (e.g., pressure from others). Similarly, Jaakkola and colleagues [17] found that students’ autonomous motivation was a significant correlate of objectively-measured MVPA during PE lessons.

PE teachers can play an important role in motivating children to be actively involved in lessons [18, 19]. Recent studies have shown that PE teachers can be trained to motivate their students more effectively and that these interventions have a positive influence on students’ motivation towards PE [20, 21]. The influence of these interventions on students’ MVPA during PE lessons, however, has received little attention [14, 22].

#### Student motivation towards PE is associated with leisure-time physical activity motivation and behavior

Student motivation towards PE lessons is positively associated with leisure-time MVPA motivation and self-reported PA outside school hours [23]. In addition, interventions with PE teachers that enhance students’ motivation towards PE, also produce increases in leisure-time physical activity intentions [21] and self-reported leisure-time MVPA [20]. There is no evidence, however, regarding the effect of interventions designed to enhance students’ motivation towards PE on objectively-measured PA outside lesson time. Given the general tendency for young people to over-report their MVPA [24] and the evidence that associations between self-reported and objectively-measured MVPA can be poor among youth [25], there is a need to examine intervention effectiveness using objective MVPA measures.

### Theoretical framework for the current study

#### Self-determination theory

Self-Determination Theory (SDT) [26] has been widely applied to a variety of life contexts, including education [27] sport [28], exercise [29] and PE [15, 16, 19, 30, 31]. According to SDT tenets, social-contextual factors (e.g., teachers’ behavior towards students) can affect individuals’ (e.g. students’) motivation by satisfying three key psychological needs: (ii) autonomy, the sense that one is acting in a self-directed manner; (ii) competence, the belief that one can interact effectively with one’s environment; and (iii) relatedness, perceptions of connectedness with significant others.

SDT also outlines two broad dimensions of motivation. Controlled motivation exists when students participate because they feel external pressure (e.g., from a teacher) or internal pressure (e.g., guilt) to participate. In contrast, students who have autonomous motivation towards PE participate because of the enjoyment or interest inherent in lessons, or because they value the outcomes of PE participation. In the context of PE, there is evidence that when teachers use motivational strategies that satisfy these three psychological needs, students experience more autonomous motivation and less controlled motivation. Importantly, autonomous motivation is positively associated with multiple adaptive outcomes [32], including student effort [19] and PA [16, 33] during lessons.

Motivational strategies that support students’ needs include, but are not limited to: (i) providing task choice and offering opportunities for students to take initiative; (ii) providing a rationale and explaining the personal relevance of an activity; (iii) acknowledging students’ difficulties when learning skills; and (iv) praising students for effort and improvement. A recent meta-analysis of randomized controlled trials (RCTs) [34], showed that these strategies can be taught effectively to a range of professionals (e.g., teachers, managers, healthcare workers), with teachers showing the greatest propensity to increase their supportive behaviors (pooled effect size *d* = 1.2). Research has also shown that using these instructional strategies is associated with greater student psychological needs satisfaction, leading to greater well-being, learning, and psychological development [19, 31, 35].

#### Previous physical education interventions based on self-determination theory

A number of recent studies have examined the effects of SDT-based interventions designed to promote needs supportive teaching in PE. For example, Tessier and colleagues [36] found that ratings by blinded observers across an eight-week period showed that teachers (*n* = 2) in the experimental condition were more needs supportive than teachers in the control condition. Another study [20] showed that teachers in the experimental condition (*n* = 5) who had been taught to use four needs supportive strategies showed greater increases in student ratings of support than teachers in the comparison condition (*n* = 5) who had only been taught two needs supportive strategies. The experimental group students also reported greater increases in autonomous motivation toward PE and more self-reported leisure-time PA than control participants. A third study [21], found that a SDT-based training intervention for teachers produced improvements in students’ needs satisfaction, autonomous motivation, and intention to be physically active during leisure time. Finally, in a study involving a single teacher, Perlman [14] found that students taught using a needs supportive teaching style were more physically active during lessons than when this teacher did not employ these strategies.

While the results of these studies are promising, a number of important limitations exist. These include: (i) evidence regarding the influence of needs supportive interventions on students’ PA during PE lessons has been limited to studies involving a small number of teachers [14] and those examining a limited range of needs supportive behaviors [22]; (ii) the lack of objective measurement of leisure-time PA, as only self-report measures have been employed [20]; and (iii) the limited assessment of the longer-term effects of these interventions [37].

### The current study

Few studies have been conducted with adolescents, but numerous studies have shown that interventions designed to improve primary and middle school PE teachers’ lesson planning and delivery can increase the MVPA that students accumulate during PE lessons [3]. These interventions have provided greater opportunities for MVPA during lessons, but little focus has been placed on enhancing students’ motivation towards PE in order to increase MVPA during lessons. Indeed, the content of many of these PA-focused interventions was designed to be enjoyable [10], but none of these interventions have been firmly grounded in relevant behavior change theory, nor have they examined whether the theoretical mediators were responsible for intervention effects on MVPA during PE lessons [3].

Theory-based interventions are needed in the PA promotion field [38–40], as they provide greater understanding of the process of behavior change and may ultimately lead to more effective interventions [41]. Self-determination theory provides a useful framework for physical activity behavior studies and an increasing number of interventions are based on SDT tenets [29, 34].

In this study, we will test a professional development intervention for teachers that combines the lesson planning and delivery approach [3] with motivational strategies designed to promote students’ needs satisfaction and autonomous motivation towards PE [20, 21]. A cluster RCT design, with allocation at the school level, was preferred to a RCT with individual level allocation because the risk of contamination effects would be high if teachers and students were randomly assigned from within a single school.

#### Web-based delivery

Previous research aimed at increasing students’ MVPA during PE has focused almost exclusively on teacher professional development conducted in face-to-face workshops [3]. While many of these interventions have produced significant and important increases in student MVPA, large-scale dissemination of exclusively ‘in-person’ professional development to teachers is logistically challenging and expensive. These challenges may constrain training to a single face-to-face workshop, which likely limits teachers’ learning and their implementation of new teaching strategies into their practice, compared with professional development delivered over an extended period of time. By capitalizing on the advantages of web-mediated professional learning, widespread reach, cost-effectiveness, and quality assurance (e.g., extending training over several sessions vs one longer session) may be achieved [42]. Thus, we designed a teacher professional development intervention that incorporates a ‘blended design’, with a mix of face-to-face and online learning.

#### Research questions

**Fig. 1:**
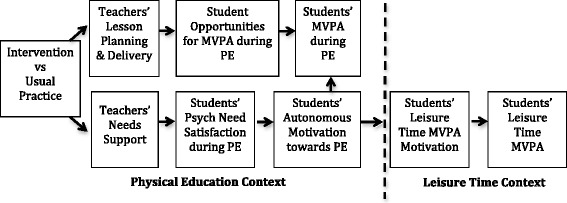
Promoting moderate-to-vigorous physical activity during physical education lessons and leisure time: a theoretical model based on self-determination theory

1. What effect does the intervention have on students’ MVPA during PE lessons (primary outcome)?
2. What effect does the intervention have on secondary outcomes?
  -  vigorous intensity PA during PE lessons,
  -  moderate intensity PA during PE lessons,
  -  sedentary time during PE lessons
  -  leisure-time MVPA, as well as moderate and vigorous physical activity measured sperately,
  -  leisure-time sedentary behavior,
  -  student needs satisfaction and motivation towards PE,
  -  student motivation towards leisure-time PA
  -  physical self-concept and subjective well-being,
  - student engagement during academic lessons following PE lessons,
3. To what extent do changes in the mediating variables (see Fig. 1) explain changes in outcomes?

#### Hypotheses

We hypothesize that, compared with students in the control condition, students whose teacher participates in the intervention will be:

1. Be more active during PE lessons (i.e., greater proportion of lesson time spent in MVPA, as well as moderate PA and vigorous PA measured separately).
2. Spend a lower proportion of PE lessons being sedentary.
3. Report greater needs satisfaction in PE, as well as higher autonomous motivation and lower controlled motivation and amotivation towards PE and leisure-time MVPA.
4. Accumulate more MVPA and less sedentary time during leisure time (i.e., outside of school).
5. More engaged in academic lessons that follow PE lessons.
6. Report greater physical self-concept and subjective well-being.

We also hypothesize that intervention effects:

7. On MVPA and sedentary behavior during PE lessons will be mediated by changes in student autonomous motivation and psychological needs satisfaction as well as teacher behavior that provides opportunities for students to engage in MVPA.
8. On MVPA behavior during leisure time will be mediated by changes in autonomous motivation towards leisure-time MVPA.
9. On student engagement in academic lessons will be mediated changes in MVPA they accumulated during the preceding PE lesson.
10.  On physical self-concept and subjective well-being will be mediated by changes in overall levels of MVPA accumulated.

## Methods

### Design

The trial will take place in government-funded secondary schools in the Western Sydney region of Australia, one of the fastest growing areas in the nation. This region has a large proportion of youth who come from low socio-economic status (SES) and immigrant backgrounds [43, 44], meaning they are at greater risk of physical inactivity compared with the Australian average [45].

**Fig. 2:**
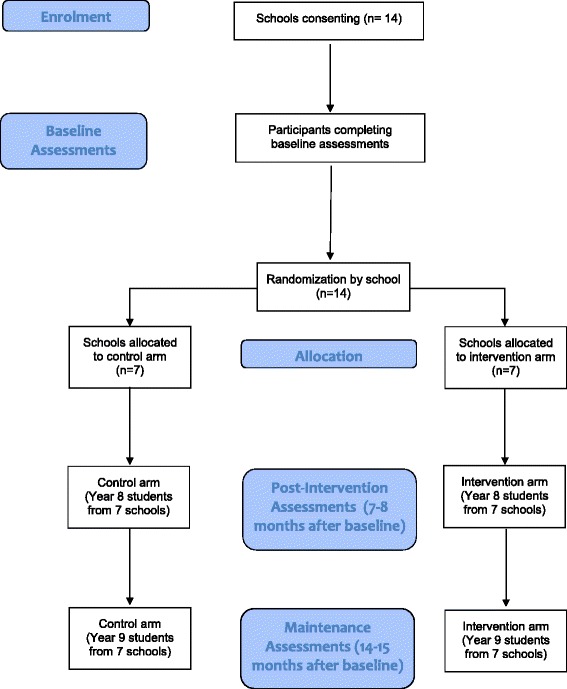
CONSORT flow diagram

This will be a two-arm, cluster randomized controlled trial with allocation at the school level (1:1 ratio to intervention and control conditions). See CONSORT flow diagram (Fig. 2). School years in Australia run from the end of January to the middle of December, with a summer break from mid-December to late January. Assessments will be completed at baseline of the first year (Term 1, February-April) and post-intervention (Term 4, September-December 2014: 7–8 months after baseline), which is the primary endpoint for this trial. A maintenance phase assessment will be conducted 14–15 months after baseline (Term 2, May–July).

### Participants

#### Schools inclusion criteria

In order to participate in the study, schools will need to meet the following inclusion criteria: (i) Secondary school with students enrolled in Years 8 and 9; (ii) Funded by the New South Wales (NSW) Department of Education; (iii) Located in the Western Sydney or Southwestern Sydney regions, as defined by the NSW Department of Education boundaries (http://www.schools.nsw.edu.au/schoolfind/locator/); (iv) Located in a postcode with low socioeconomic status, as defined by a decile rank of ≤ 5 according the Australian Bureau of Statistics’ Index of Relative Socioeconomic Disadvantage; and (v) Permission granted by the Principal, the Head Teacher of PE and at least one Year 8 PE teacher.

#### Teachers inclusion criteria

All PE teachers in participating schools, not only those who teach Year 8 during the main portion of the intervention, will be eligible to participate in the intervention. Including teachers from other year groups is intended to help ensure that when students move to Year 9 during the maintenance phase of the study, they will be taught by teachers who have completed the intervention training. Teachers who join the PE staff after the intervention will be invited to attend specially scheduled face-to-face sessions during which content will be taught.

#### Student inclusion criteria

All students enrolled in Year 8 PE classes in participating government-funded (NSW Department of Education) secondary schools in the Western Sydney region of NSW. Parental consent and student assent to participate will be obtained.

#### Student exclusion criteria

Students unable to participate in PE lessons (e.g., due to medical issues). Students who do not provide assent or did not receive parental consent to participate in the study will be excluded from assessment procedures, but will participate in PE lessons.

Human research ethics approval has been obtained from the Western Sydney University (H9171), Australian Catholic University (2014185 N) and the NSW Department of Education (2013162).

### Sample size

Sample size calculations were based on estimated effect sizes for the primary outcome. A recent meta-analysis [3], indicated that lesson planning and delivery interventions have a pooled effect of *d* = 0.60 on MVPA during PE lessons. To ensure 80 % power to detect an effect of this size would require 90 participants in a non-clustered trial (two-tailed probability level of 0.05). We considered the clustered nature of the data, including both school and class levels [46]. However, results from recent studies of MVPA in PE lessons indicated that clustering at the school level was negligible after accounting from clustering at the class level [22, 33]. As a result, we adjusted our sample size calculations for clustering using the formula 1 + (*m*-1)ρ, where *m* is the number of students per class and ρ is the intra-class correlation (ICC) [46].

Our recent study in Sydney-area schools indicated a class-level ICC of 0.62 [22], which was similar to Aelterman et al. [33] who reported a class-level ICC of 0.63. With an estimated class size of 22 participating students, an adjustment of 14.23 was required: 1 + (22–1)*0.63 = 14.23. Multiplying by the 90 participants required in non-clustered trial, we estimated that 1280 students would need to participate to achieve 80 % power. We estimate that 14 schools, with a mean of 4.5 classes of Year 8 students per school can be recruited and, therefore, aim to recruit 1386 students (14 schools × 4.5 classes per school × 22 students per class). Potential loss to follow up of up to 30 % of participants (*n* = 384; a very conservative estimate) will not substantially impact on the statistical power of the study. This resilience to loss of power exists because this study will use generalized mixed models, enabling the inclusion of all participants (included at baseline) in the analyses. In non-clustered longitudinal clinical trials, this analytic approach has been shown to yield similar point estimates and smaller decreases in statistical power than multiple imputations [47, 48].

### Blinding

Trained research assistants who will be blinded to school allocation will conduct baseline, post-intervention and maintenance phase assessments. Students participating in the study will also be blinded to hypotheses and school allocation. Teachers will be aware of their allocation to the intervention or control condition.

### Recruitment and randomization

Principals and Head Teachers of PE from all secondary schools that meet our eligibility criteria will be invited to express their interest in participating in this study. From the schools that indicate an interest in the study, we will attempt to purposively select schools in order to ensure that the sample is representative of the region’s population, in terms of school size and gender composition (i.e., single sex or co-educational). Schools that agree to participate will be match paired according to SES of the postcode in which the school is located (according to the Index of Relative Socio-Economic Disadvantage:), school size (Year 8 enrolment), gender composition of PE classes (i.e., single sex vs co-ed) and the duration of PE lessons. Using a computer-based randomization plan generator, the 14 schools will be randomized to the control or intervention condition from within each pair following baseline assessments. A researcher not associated with recruitment or data collection, and who will be blind to school identity, will carry out randomization procedures.

All PE teachers in schools that enter the trial will be invited to an information session during which study procedures will be outlined and questions answered. The research team will present study information and provide an information letter and consent form to all Year 8 students from classes whose teacher agrees to participate.

### Intervention

#### Overview

An overview of the intervention can be found in Table 1. During the main portion of the intervention (six months: Terms 2 and 3 of 2014), teachers will participate in two days of face-to-face workshops at a local university and complete two implementation tasks at their school. These implementation tasks will involve video-based self-reflection via the project’s Web 2.0 platform and an individualized feedback meeting with a project mentor. Each intervention school will also complete two group peer-mentoring sessions at their school (one per term) in which they will discuss implementation with members of their school PE staff.

In the booster period (Term 1 of 2015), teachers will complete a half-day face-to-face workshop at their school, plus one implementation task and a group mentoring session at their school. Throughout the entire intervention period (main intervention plus booster period), teachers will have access to online resources, including videos of good/poor practice, sample lesson plans and a discussion forum. The NSW Board of Studies, Teaching and Educational Standards has accredited this training.

#### Main intervention

##### Face-to face professional development workshops for teachers

During the main intervention period (Terms 2 and 3, Year 1), training will involve two days of face-to-face workshops, including presentations by members of the research team (CL and DL), videos of best/poor practice examples viewed on the project website, video-based self-reflection via the project website, group discussion, opportunities for teachers to practice implementing taught principles in simulated scenarios, and action planning (i.e., goal-setting) via the project website. All workshops will take place at Western Sydney University, where key facilities include a large classroom and a gymnasium. A complete listing of workshop components can be viewed in Additional file 1: Appendix A.

Workshop training will have two broad aims: (i) to teach teachers motivational strategies that can be implemented in their PE lessons; and (ii) to help teachers plan and deliver lessons that maximize opportunities for MVPA.

Lesson planning and delivery strategies are based on interventions previously employed in large-scale trials mostly in the United States [10, 11, 49]. Briefly, these strategies are designed to create teacher awareness of PA and sedentary behavior levels in their lessons, minimize management and instruction time, and maximize opportunities for PA [10]. Motivational strategies are based on SDT tenets. The goal of this portion of the training is to teach PE teachers how to support their students’ basic psychological needs during lessons, thus promoting autonomous motivation towards PE and leisure-time PA. Details of all AMPED strategies can be viewed in Table 2.

**Table 1:** Overview of intervention components

Phase	Component	Timing
Main Intervention (Year 1)	Face-to-Face Workshop 1 (1 day)	Start of Term 2, May
	Technology-assisted Implementation Task 1	Term 2, May–June
	Group Mentoring Session	End of Term 2, June
	Face-to-Face Workshop 2 (1 day)	Start of Term 3, July
	Technology-assisted Implementation Task 2	Term 3, July–September
	Group Mentoring Session	End of Term 3, September
Booster Intervention (Year 2)	Face-to-Face Workshop 3 – Booster (1/2 day)	Start of Term 1, February
	Technology-assisted Implementation Task 3	Term 1, February–March
	Group Mentoring Session	End of Term 1, March

**Table 2:** AMPED Intervention principles and teaching strategies

AMPED Principles	AMPED Teaching strategies
Maximising Movement and Skill Development	1. Include an active warm-up with dynamic stretching.
	2. Provide lots of equipment.
	3. Employ circuits and rotations.
	4. Use grids effectively.
	5. Implement small sided games.
	6. Organise non-elimination games.
	7. Modify games to maximize activity and skill development.
	8. Integrate fitness into activities.
	9. Choose activities that maximize MVPA.
Reducing Transition Time	1. Manage the change room effectively.
	2. Take the roll while students are active.
	3. Early activity set-up.
	4. Distribute equipment quickly.
	5. Decrease talk/instructions.
	6. Form groups efficiently.
	7. Manage water breaks efficiently.
Building Competence	1. Provide overview of lesson/unit.
	2. Make behavioural expectations clear.
	3. Use questioning.
	4. Provide effective positive feedback.
	5. Provide effective corrective feedback.
	6. Match task to ability level.
	7. Limit peer comparison.
	8. Promote self-comparison.
Supporting Students (including support for students’ autonomy and relatedness needs)	1. Emphasise fun and variety.
	2. Circulate around the class.
	3. Provide students with opportunities to make choices.
	4. Provide a rationale and emphasise relevance.
	5. Minimise controlling language and behavior.
	6. Take the students’ perspective.

Definitions for each principle and strategy can be found in Additional file 1.

**Fig. 3:**
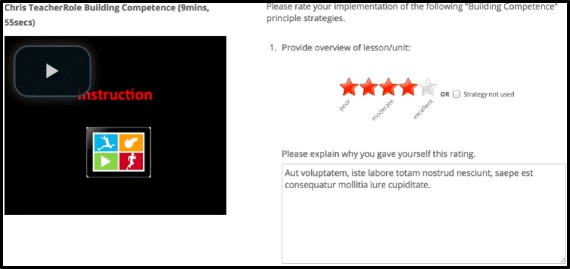
Screen shot of a self-reflection exercise on the AMPED website.

Importantly, many aspects of the face-to-face workshop will be delivered using web-based resources. For example, best/poor practice videos will be accessed through the project website during face-to-face workshops. Teachers will also conduct their first self-reflection task during the workshop by accessing videos of their lessons that will be uploaded onto the project website. Teacher self-reflection will involve a rating of their implementation of each AMPED strategy (1 to 5 stars) and free-text response explaining why they provided this rating. See an example in Fig. 3. Additional file 1: Appendix B provides details of all ratings and descriptions. Thus, for much of the workshop, teachers will work independently on the web platform, but project staff will be available to provide assistance at any stage.

All teachers in the intervention condition will be provided with a touchscreen tablet computer (iPad) to use during the workshops and implementation in their schools. Using this technology will allow us to blend learning during the face-to-face workshops with implementation tasks completed in schools. It is important to provide this equipment to teachers as pilot work in local schools showed that many computers in NSW Department of Education schools operate outdated versions of web browsers that do not support the project’s website. Many teachers suggested that updating computer software would be a significant barrier to implementation, as it required a school network administrator to complete the process.

##### Technology-assisted implementation in schools

Teachers in the intervention condition will have access to the project’s interactive website. The site guides teachers through a series of three implementation tasks, with one task to be completed after each face-to-face workshop (two during main intervention, one during booster intervention).

The first step in each implementation task will require teachers to set an ‘action plan’ (i.e., goals) for implementation of AMPED strategies taught during the face-to-face training workshop into their lessons during the month following the workshop. The second task will require teachers to reflect on their implementation of AMPED strategies, using video clips of their teaching during the month following the workshop. Specifically, project staff will film a scheduled lesson occuring after the workshop, which will then be analyzed using a coding system designed to identify events that take place during lessons and are relevant to principles taught during the face-to-face workshop. For example, each time a teacher provides students with feedback during a lesson, this event will be ‘tagged’ and a short video clip containing all instances of teacher feedback will be uploaded to the website. Teachers can then reflect on the extent to which this feedback aligns with strategies designed to support students’ psychological needs (e.g., Fig. 3).

The third and final step in the implementation task will involve a mentoring conversation at each teacher’s school. Following each self-reflection task, teachers will meet with one of three mentors who are part of the project team. A mentor will review each teacher’s video clips and then facilitate a feedback conversation with the teacher. This conversation will be the final step in the first implementation task. Mentors will then help teachers to set a new action plan for implementation over the remainder of the term.

Project mentors will have at least three years of experience teaching PE and will complete two days of training designed to ensure in-depth understanding of AMPED intervention principles. This training will involve reading and discussing SDT-related research papers, learning about the mentors’ role and participation in the intervention, and practice using the video coding system and website for viewing mentees’ videos. The peer mentors will also practice their mentoring skills in mock mentoring sessions, reflect on their own mentoring practice and provide feedback on the other peer mentors’ performance.

Teachers will complete the implementation task three times-once following each workshop. They will also participate in three group mentoring conversations at their school-once per term during the two main intervention terms and once during the booster period. These group mentoring sessions will take place at each school and involve PE staff members from that school. The purpose of these sessions is to encourage staff to reflect on their implementation of AMPED strategies and devise methods to support each other’s implementation in the future. A project mentor will lead the first group mentoring session. A member of each school’s PE staff will lead the second mentoring session, with the project mentor observing and providing the staff member with private feedback following the session. The staff member will lead the third and final group mentoring session on his/her own during the booster period.

In addition to facilitating teachers’ implementation tasks, the project website will provide teachers with resources designed to facilitate successful implementation of principles from the face-to-face workshops. The site utilizes a Web 2.0 platform that allows for dynamic interaction between the teachers and the research team. For example, teachers will be able to rate and comment on each of the resources that the research team posts on the site. This teacher feedback will automatically update the list of top rated resources on the site. Resources will also be ‘pushed’ to teachers when they create each action plan. Specifically, each resource will be linked via a database to relevant AMPED strategies. Thus, when a teacher includes an AMPED strategy as part of an action plan, an email will be sent to the teacher with links to relevant resources that will aid implementation.

The web platform also includes a community of practice discussion forum where teachers can share experiences from their attempts to implement specific strategies from the intervention in their own PE lessons. The project manager, with input as required from the project mentors and lead investigators, will moderate the discussion and provide ongoing advice to help teachers overcome barriers to implementation.

**Fig. 4:**
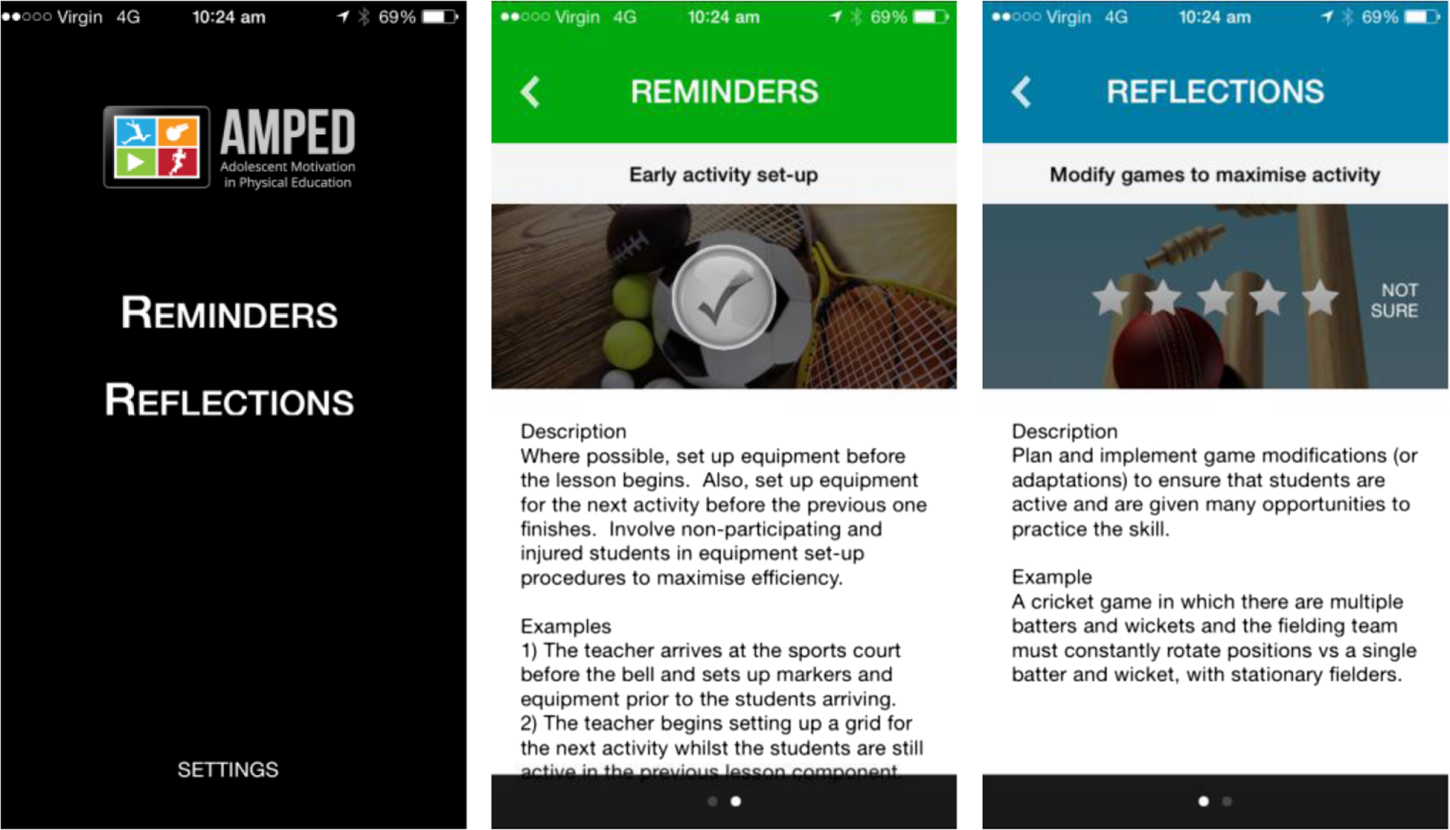
Screen shots of the AMPED mobile application.

Teachers will also be able to download an AMPED mobile application (‘app’) that provides reminders to implement their chosen strategies at an interval of their choosing (range = once per day to once every eight weeks). The app will also prompt teachers to conduct brief self-reflections on their implementation of AMPED strategies, with the same interval range as the reminder function. See Fig. 4 for screenshots from the app.

##### Physical activity feedback for teachers

During the second workshop, teachers will be provided with private, individualized information regarding their students’ MVPA levels during lessons at baseline (as measured via accelerometry). In their action plan near the end of the workshop, teachers will be asked to set a goal for MVPA during lessons in the upcoming term. In the first four weeks following the workshop, a research assistant will visit each teacher’s class and fit students with accelerometers for one lesson. These data will be processed and mentors will provide teachers with feedback regarding student MVPA levels during their individual mentor meetings later in the term.

#### Booster intervention

Five months after the end of the intervention, mentors will meet with experimental group teachers for a half-day face-to-face workshop in each school. The purpose of this meeting is to reflect on the core principles of (i) lesson planning and delivery and (ii) motivational support from the first two workshops. Teachers will be encouraged to reflect and discuss their attempts to improve their practice from the previous year. Each teacher will be asked to create an action plan for the upcoming term and use the website or mobile app to reflect on his or her implementation by the middle of the term. In the final weeks of the term, each school will be asked to hold a final group mentoring meeting. A member of the school’s PE staff will facilitate this meeting; project mentors will not be present. Teachers will be able to access the project website and the mobile app throughout the rest of the study, including the maintenance phase assessment.

Teachers in the control condition will not receive training during the main RCT, but will be offered this opportunity in the period following the trial.

#### Compensation

All teachers who complete AMPED training will receive 23 registered hours of NSW Board of Studies, Teaching and Educational Standards teacher professional learning credits at no charge; NSW teachers must complete 50 hours of registered teacher professional learning every five years to maintain their accreditation. Teachers will also keep their touchscreen tablet computer that will be provided to facilitate interaction with project website. Finally, at each data collection time point, teachers will receive a $50 gift card to compensate them for time spent organizing student consent and facilitating data collection scheduling, a task that is beyond their usual teacher duties. All of this compensation will be provided equally to teachers in the intervention and wait-list control schools.

### Fidelity

Three lessons from each class in the experimental and control conditions will be video- and audio-recorded during baseline, post-intervention, and maintenance phase assessment periods. The video camera will be located at an angle that allows the entire lesson to be recorded. Audio recordings of the lesson will be captured using a wireless microphone. Trained, blinded observers will rate a random sample of 10 % recordings (approximately 60 of 600 lessons) to assess fidelity of implementation. Intervention fidelity will be confirmed if, compared with controls, the intervention condition’s lessons during the post-intervention and maintenance phases show greater implementation of teaching strategies taught during the intervention, including (i) lesson planning/delivery strategies designed to maximize opportunities for physical activity during lessons and (ii) strategies designed to support student needs satisfaction and foster autonomous motivation. See Table 2 for a full listing of AMPED strategies and Additional file 1: Appendix B for details on the rating protocol.

### Outcomes

Trained research assistants will collect all outcome data, including behavioral outcomes, psychological outcomes and educational outcomes. To ensure consistency and enhance data quality, these researchers will follow standard operating procedures (as outlined in a protocol manual) before, during, and after all data collection sessions. Questionnaire data will be collected in regularly scheduled classroom lessons, supervised by two research assistants who will follow a ‘read-aloud’ protocol for questionnaire instructions and items.

#### Primary outcome

MVPA during PE lessons-ActiGraph accelerometers (ActiGraph, LLC, Fort Walton Beach, FL) will be used to measure PA during three PE lessons at each time point (baseline, post-intervention, and maintenance). Accelerometer models will include GT1M, GT3X, and GT3X+, which have been shown to have extremely high levels of agreement both in terms of counts per minute and MVPA estimates [50]. Accelerometers will be attached using cotton, elastic belts to the students’ hip at the top of the right iliac crest.

Lesson start and end times will be signified by the school bell and will be recorded by researchers on site. These times will be used to process accelerometer data and determine PA that occurs during each PE lesson.

Prior to data collection, accelerometers will be initialized and set to record data using a 1 sec epoch or 60hz (for GT3X+). Data will be processed using Actilife software (Version 6, ActiGraph, LLC, Fort Walton Beach, FL), with 1 sec vertical axis data used to classify activity intensity according to Evenson and colleagues’ [51] cutpoints, which have been shown to be the most accurate in this age group [52]: moderate-to-vigorous activity >2296 counts per minute. To convert the cutpoint definition for 1 s epochs, the cut-point will be divide by 60.

#### Secondary outcomes

##### Behavioral outcomes

Sedentary behavior and light, moderate and vigorous physical activity during PE lessons-In addition to MVPA during PE lessons, accelerometer data will be used to examine the amount of lesson time spent in sedentary behavior and light, moderate and vigorous physical activity: vigorous activity (>4012 counts per minute), moderate activity (2296–4011 counts per minute) and light intensity activity (101 – 2295 counts per minute). Sedentary behavior will be classified as ≤100 counts per minute.

Student participation rate in PE lessons-Research assistants will record the total number of students at each lesson, the number of students at each lesson that are not participating and the number of students absent from each lesson. These data will be used to calculate the proportion of students’ participating in each lesson.

Physical activity and sedentary behavior during leisure time (i.e., outside school)-Students will be instructed to wear their accelerometer for five weekdays and two weekend days at each time point (baseline, post-intervention, and maintenance). Accelerometer data processing procedures (e.g., activity intensity classification) will be consistent with those described for PE lessons. Periods of non-wear time will be defined as 30 minutes or more of consecutive ‘0’ counts and will be removed from the dataset. This non-wear criterion was chosen because research with adolescents indicates that 30-minute bouts of sedentary behavior (≤100 counts per minute) are extremely rare [53]. To be included in the analyses, the student will need to provide valid data for at least three days in total, including at least two weekdays. Valid days will be defined as days with ≥ 8 h of wear time. These criteria have been chosen because: (i) compared to more stringent criteria, they are likely to minimize missing data, which is important for maintaining population representativeness in this repeated-measures group randomized trial [54, 55]; and (ii) they are relatively consistent with other accelerometer studies in adolescents [56]. To ensure increased compliance with this protocol we will: (i) deliver thorough, clear, and standardized initial instructions during a classroom lesson; (ii) send an SMS text message to students each morning reminding them to put the accelerometer on; and (iii) send an SMS text message prior to the day of accelerometer collection to ask teachers to give a verbal reminder to students regarding wearing and return. Leisure time MVPA will also be measured subjectively using an adaptation of two versions of the Health Behavior in School-Aged Children questionnaire [57, 58].

##### Psychological outcomes

Students’ physical self-concept-Aspects of students’ global and physical self-concept will be measured using 21 items from the Physical Self Inventory. This scale includes items to measure: (i) global self-worth; (ii) physical self-worth; (iii) physical appearance; (iv) sport competence; (v) physical condition; and (vi) physical strength [59].

Students’ subjective well-being-The Positive and Negative Affect Scale for Children will be used to measure subjective well-being. This scale has produced reliable and valid scores in youth samples [60].

##### Educational outcomes

Student engagement in academic lessons-Students’ self-reported engagement during mathematics lessons will be measured using a questionnaire that was originally designed to measure school engagement [61], and later adapted to a mathematics setting [62]. The questionnaire aims to assess students’ cognitive, behavioral and affective engagement. At each time point, students will be asked during a mathematics lesson to report on their typical engagement with mathematics (e.g., “I like maths lessons”). On a different day, students’ will also report on their level of engagement during that specific lesson (e.g., “Today, I liked the maths lesson”).

On-task behavior during academic lessons—We will also video record students’ mathematics lessons. These recordings will be analyzed using a momentary time sampling procedure to determine students on-task behavior. This procedure has been successfully implemented in investigations conducted by others [7]. Analysis of observations will take place from data gathered at baseline and post-intervention. Within lessons, students will be chosen at random and observed in intervals for the duration of the lesson. Students will be observed and categorized as being either on or off task. Additionally, on-task behavior will be further coded as actively engaged or passively engaged. Intra- and inter-observer reliability will be established before the assessment period.

Student academic performance – will be measured by collecting National Assessment Program Literacy and Numeracy (NAPLAN) scores from records held by the NSW Department of Education.

#### Motivational mediators

Students’ perceptions of teachers’ needs supportive and controlling behaviors-Students’ perceptions of their PE teacher’s involvement (i.e., relatedness support), structure (i.e., competence support) and autonomy support will be measured using 12 items from the Teacher as Social Context Questionnaire [63]. Student perceptions of controlling behavior will be measured using the intimidation and conditional regard subscales of the Controlling Interpersonal Style Scale (adapted to suit the PE context) [64].

Psychological Needs Satisfaction during PE lessons-Autonomy [65], competence [66], and relatedness [67] need satisfaction scales. These measures have produced strong reliability and validity evidence in adolescent samples [19].

Autonomous motivation, controlled motivation and amotivation towards PE-Perceived Locus of Causality Questionnaire [68]. Scores derived from this questionnaire have shown good reliability and validity in multiple adolescent samples [69].

Autonomous motivation, controlled motivation and amotivation towards leisure-time PA-Behavioral Regulation in Exercise Questionnaire-2 [70]. This measure has produced reliable and valid scores in studies involving adolescent samples. [71].

#### Demographic information

Students will report their country of birth and language spoken at home. Using categories based on the Australian Bureau of Statistics’ Standard Classification of Languages [72], we will use language data to categorize students into one of seven ethnic backgrounds (English, European, Middle Eastern, Asian, African, South Pacific, or ‘other’). Students will also indicate if they are of Indigenous origin (i.e., Australian Aboriginal or Torres Straight Islander). We will measure the socioeconomic status of each participant’s family using the Family Affluence Scale [73].

Research assistants will measure students’ height to the nearest 0.1 cm using a portable stadiometer (Surgical and Medical Products No. 26SM, Medtone Education Supplies, Melbourne, Australia). They will also measure students’ weight to the nearest 0.1 kg using digital scales (UC-321, A&D Company LTD, Tokyo, Japan). These data will be used to calculate each student’s body mass index (BMI) and BMI Z-score [74].

#### Covariates-related to MVPA during PE lessons

Temperature at the start of the PE lesson will be measured using an electronic thermometer (HC520 Thermometer, CS Raffles Tech Inc., Singapore). The type of activity being taught during the lesson will also be recorded and categorized as follows: (i) invasion games; (ii) striking and fielding games; (iii) target games; (iv) net/wall games; (v) fitness activities; (vi) artistic activities; (vii) athletics; (viii) other; and (ix) mix of activities. Finally, at some lessons students will arrive wearing an accelerometer that they have been provided in order to measure their daily MVPA (7-day wear period). For other lessons, students will be fitted with an accelerometer when they arrive at the lesson. The timing of this fitting (i.e., do they arrive at the lesson wearing an accelerometer or not?) will be recorded for each student at each lesson.

### Statistical analysis

Between-arm differences in changes on the primary and secondary outcomes will be analyzed using generalized linear mixed models according to intention-to-treat and per-protocol principles. Specifically, intention to treat analyses will involve analysis of outcomes collected from all students who completed baseline assessment, regardless of whether or not they complete post-intervention or maintenance phase assessments. Between-arm differences in changes on the primary and secondary outcomes will be assessed by including an indicator variable for group allocation (arm), a variable representing time (baseline, post-intervention, and/or maintenance) and their interaction.

For the primary outcome (MVPA during lessons), student data will be gathered from between one and three lessons per student at each of the time points (baseline, post-intervention and maintenance). The repeated measure will be modeled as a random intercept effect. Lesson will also be modeled as a random intercept effect. Analyses will also be adjusted for class and teacher clustering-each teacher will be given a case ID. In instances where teachers ‘team-taught’ (i.e., two teachers within a single lesson), we will create a new case ID that is unique to that combination of teachers. In some instances students will be taught by different teachers within a time point (i.e., from lesson to lesson) or across time points (e.g., from baseline to post-intervention). We will account for this data structure by specifying cross-classified random effects models. Possible school-level residual clustering will be explored and included in the models, if appropriate. In sum, we will include four random intercept effects: (i) lesson; (ii) student; (iii) teacher; and (iv) class. We will consider a fifth random intercept effect for school, if preliminary analyses suggest clustering of MVPA during lessons at the school level.

We will also control for a number of variables that could influence students’ MVPA during lessons. These include temperature at the start time of the lesson, the type of activity included in the lesson and the timing of accelerometer fitting for the lesson (the student arrived at lesson wearing an accelerometer or was fitted at started of lesson).

Generalized linear mixed models will be also used to examine mediation hypotheses. Mediating effects will be estimated using a cluster-bootstrapped based product-of-coefficients test that is appropriate for cluster randomized controlled trials [75].

Potential moderators of the intervention effects will also be explored. Potential moderators will include gender and ethnic background (categorical variables), as well as socio-economic status and baseline levels of physical activity and psychosocial variables (e.g., motivation and needs satisfaction) which will be treated as continuous variables. Moderator effects will be explored using the same generalized mixed modeling approach by including appropriate interaction terms in the regression models. The trial is powered to detect main effects, not interactions; thus, we will employ a less stringent significance level to explore potential moderators. Interaction terms that are significant at *p* < 0.1 will be explored by testing differences in intervention effects across sub-groups stratified according to the moderator variable [76].

Per protocol analyses will investigate the influence of teachers’ adoption of the intervention, as indicated by the proportion of intervention components completed by each teacher (e.g., workshops attended and online tasks completed), on student outcomes (e.g., MVPA during PE lessons). Analyses will also examine the effect of teachers’ implementation of the intervention, as indicated by increases in their use of AMPED teaching strategies from baseline to post-intervention (observed in video recordings of their lessons), on student outcomes.

## Discussion

The purpose of this study is to evaluate an intervention designed to increase the amount health-enhancing PA that secondary school students accumulate during their school-based PE lessons. The intervention brings together, for the first time, strategies designed to maximize opportunities for students to be active with strategies intended to enhance their motivation for PA. The intervention is delivered, in part, using an online training platform for teachers that will help facilitate more widespread dissemination compared with interventions that rely exclusively on face-to-face training.

## Additional file

Additional file 1:
**Appendix A**. Workshop schedules. **Appendix B**. Video rating guide for teachers and independent observers (DOCX 978 kb)

## Abbreviations

AMPED: Activity and Motivation in Physical Education
BMI: body mass index
ICC: intra-class correlation
MVPA: moderate-to-vigorous physical activity
NSW: New South Wales
PA: physical activity
PE: physical education
RCT: randomized controlled trial
SDT: self-determination theory
SES: socioeconomic status
