# Supplementary material for: An Internet-supported Physical Activity Intervention Delivered in Secondary Schools Located in Low Socio-economic Status Communities: Study Protocol for the Activity and Motivation in Physical Education (AMPED) Cluster Randomized Controlled Trial
Source: BMC Public Health. 2016 Jan 6;16:17. doi: 10.1186/s12889-015-2583-7 (PMC4704292; doi:10.1186/s12889-015-2583-7)
Supplement: Additional file 1: — Appendix A. Workshop schedules. Appendix B. Video rating guide for teachers and independent observers (DOCX 978 kb) [file 12889_2015_2583_MOESM1_ESM.docx]

**Appendix A – Workshop Schedules**

**Workshop 1 - Overview**

| **Activity** | **Time** |
| --- | --- |
| **Welcome** | 8.15-8.45 |
| **Overview of the Project** | 8.45-9 |
| **Principle 1 -Maximising Movement and Skill Development (MMSD)**  Lecture on evidence-based strategies (30 min)  Good/poor practice video reflection on tablet (30 min)  Group discussion and coffee (30 min)  ‘My practice’ self-reflection on tablet (30 min) | 9-11 |
| **Brief microteaching**  Targeting 2-3 MMSD strategies that based on observations from baseline are not being implemented well in lessons  Structured discussion on these 2 to 3 strategies | 11-12 |
| **Lunch** | 12-12.30 |
| **Principle 2 – Building Competence (BC)**  Lecture on evidence-based strategies (30 min)  Good/poor practice video viewing and reflection using tablet (30 min)  Group discussion and coffee (30 min)  My practice self-reflection using tablet (30 min) | 12.30-2.30 |
| **Micro Teaching 2**  Targeting 2 or 3 BC strategies | 2.30-3.15 |
| **Action Plan & Resources**  Introduce action plan concept and resources.  Each teacher completes an action plan on his/her tablet - facilitated by mentor.  Prompt teachers to explore the resources section for ideas. | 3.15-3.45 |

**Note:** Teachers from multiple schools will attend Workshop 1 at the university campus.

**Workshop 2 - Overview**

| **Activity** | **Time** |
| --- | --- |
| **Welcome**  Overview  Feedback on Term 1 physical activity  Mentor’s observations on Term 2 teaching | 8.15-9 |
| **Principle 3 –Supporting Students (SS)**  Lecture on evidence-based strategies (30 min), with examples of great practice from Term 2  Good/poor practice video reflection (30 min)  Group discussion and coffee (30 min)  My practice self-reflection on tablet (30 min) | 9-11 |
| **Brief microteaching**  Target 2-3 SS strategies  Structured discussion on 2 to 3 SS strategies | 11-12 |
| **Lunch** | 12-12.30 |
| **Principle 4 – Reducing Transition Time**  Lecture on evidence-based strategies (30 min), with examples of great practice from Term 2  Good/poor practice video viewing and reflection (30 min)  Group discussion and coffee (30 min)  My practice self-reflection using tablet (30 min) | 12.30-2.30 |
| **Action Plan**  Download and review mobile app functions   - Review action plan portion of the website.   Each teacher completes an action plan on his/her tablet - facilitated by mentor. | 2.30-3pm |

**Note:** Teachers from multiple schools will attend Workshop 2 at the university campus.

**Workshop 3 - Overview**

| **Activity** | **Time** |
| --- | --- |
| 1. **Welcome and Introduction** | 12.00-12.10 |
| 1. **Review**  - Project mentor reviews AMPED principles and prompts discussion of strategy implementation stories from teachers | 12.10-1.50 |
| 1. **Break** | 1.50-2.05 |
| 1. **Brainstorming for implementation**  - Project mentor facilitates discussion of ways to implement AMPED strategies | 2.05-2.40 |
| 1. **Action Plan**   Each teacher completes an action plan on his/her tablet - facilitated by mentor. | 2.40-3.00 |

**Note:** Workshop 3 takes place in each of the 7 intervention schools. Only teachers from each host school will attend each workshop.

**Appendix B – Video Rating Guide for Teachers and Independent Observers**

Below is the rating guide that will be provided to teachers during Workshops 1 and 2. Blinded, independent observers will also use this guide to evaluate teachers’ implementation of the strategies in video recordings of lessons at baseline and post-intervention.

**
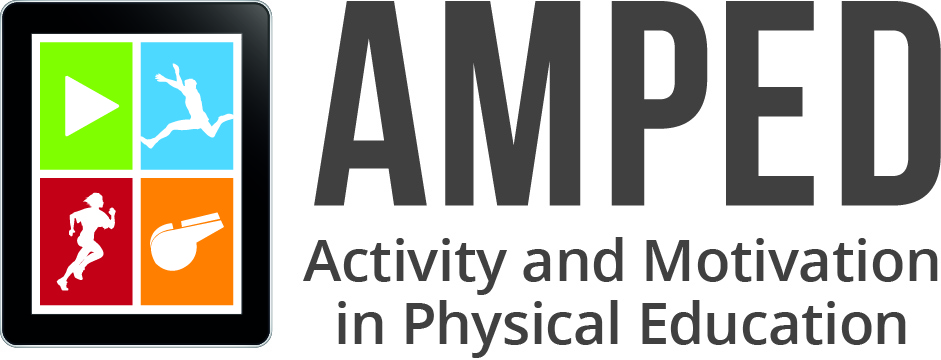
**

**Teachers’ Guide for Rating the Quality of Strategy Implementation**

Below is a 5-point likert scale that will help you determine the extent to which you have implemented each strategy during a recent lesson that we video recorded for you.

On the ‘**My Practice’ page of the AMPED website’s reflection section** (see screenshot below), please use this scale to rate the ***quality*** of your implementation of ***each*** strategy.

| ***1*** | ***2*** | ***3*** | ***4*** | ***5*** |
| --- | --- | --- | --- | --- |
| ***Poor*** |  | ***Moderate*** |  | ***Excellent*** |

**OR** If you did not implement the strategy at all, please select “strategy not used”.

***
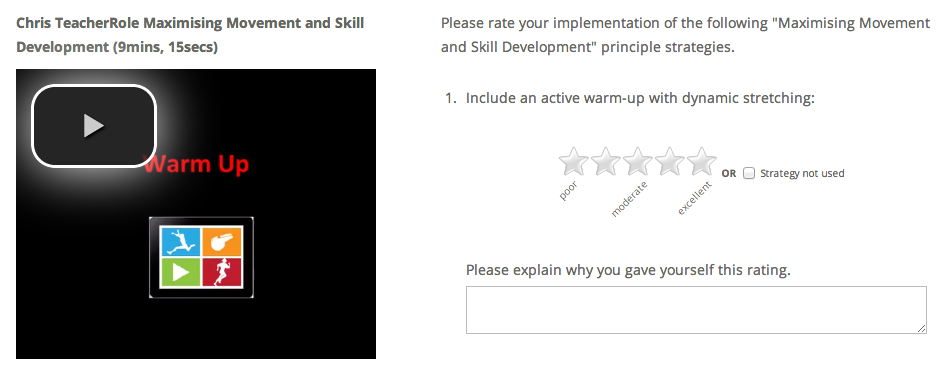
***

***On the following pages you will find descriptions and examples that will help you rate the implementation quality of each strategy.***

**Principle 1: Maximising Movement and Skill Development**

**Strategy 1: Include an Active Warm-up with Dynamic Stretching**

**Description:** *Plan and implement a warm-up activity that includes dynamic stretching and gives all students the opportunity to be active. This may involve opportunities for movement skill practice.*

***Example:*** *A tag game that allows all students to be active for the entire warm-up (no elimination) and provides time for dynamic stretching to occur vs traditional Rob the Nest (a tag game that involves short bursts of activity, and longer periods of sedentary time) followed by static stretching.*

| **Characteristics of *Poor*** | ***Characteristics of Moderate*** | **Characteristics of *Excellent*** |
| --- | --- | --- |
| Many students are sedentary (standing/sitting/lying down) for substantial portions of the warm-up. | Some students are sedentary for substantial portions of the warm-up. | Most or all students are active for the majority of the warm-up. |
| Static stretching. | Mix of static and dynamic stretching. | Dynamic stretching. |
| Warm-up requires little or no skill development (e.g., running laps around a sports court). | Warm-up requires students to engage in a small amount of skill development (e.g., fundamental movement skills or activity-specific skills). | Warm-up requires students to engage in substantial skill development (e.g., fundamental movement skills or activity-specific skills). |

**Strategy 2: Provide Lots of Equipment**

**Description:** *Plan and implement activities that use many pieces of equipment to ensure students practice skills more often (e.g., more touches).*

***Example:*** *Students practicing their soccer passing skills in small groups versus students practicing in whole class games (e.g., one ball per class in soccer).*

| **Characteristics of *Poor*** | ***Characteristics of Moderate*** | **Characteristics of *Excellent*** |
| --- | --- | --- |
| The amount of equipment provided is minimal throughout the lesson, leading to long intervals between skill attempts. | Some activities have lots of equipment, but others activities have limited equipment. | Many pieces of equipment provided for all activities leads to frequent skill attempts. |

**Strategy 3: Employ Circuits and Rotations**

**Description:** *Plan and implement circuits and rotations to ensure that students are active and are given many opportunities for skill development.*

***Example:*** *A fitness measurement lesson may include a number of stations and students rotate vs a lesson in which all students wait their turn to be measured on each test.*

| **Characteristics of  *Poor*** | ***Characteristics of  Moderate*** | **Characteristics of  *Excellent*** |
| --- | --- | --- |
| All students complete the same activity at the same time. | More than one activity occurs at a time, but organisation is poor and students spend too much time waiting for their turn. | Students move from station to station completing different activities at the same time or there are highly active stations in a circuit. |

**Strategy 4: Use Grids Effectively**

**Description:** *Plan and implement grids to ensure that students are active and are given many opportunities to practice the skill.*

***Example:*** *Using grids, students practice in small groups to complete successful throws and catches vs pair throwing and catching in two lines.*

| **Characteristics of  *Poor*** | ***Characteristics of  Moderate*** | **Characteristics of  *Excellent*** |
| --- | --- | --- |
| Students participate in activities that involve very limited movement and skill practice and / or large wait times between skill practice. | Students participate in grid activities that sometimes involve movement and skill practice. | Students participate in grid activities that usually or always require movement and skill practice. |

**Strategy 5: Implement Small Sided Games**

**Description:** *Plan and implement small-sided games to ensure that students are active and are given many opportunities for skill development*

***Example:*** *2 games of 5 v 5 half-court basketball with a few students waiting and practicing skills off the court vs a single full-court game of 5 v 5 basketball with more than half the class watching from the side.*

| **Characteristics of  *Poor*** | ***Characteristics of  Moderate*** | **Characteristics of  *Excellent*** |
| --- | --- | --- |
| Entire class participates in a single game. | The class is divided into at least two groups to increase student activity and skill attempts. | Small-sided games or activities are employed effectively so that students are active most of the time and have many opportunities for skill development. |

**Strategy 6: Organise Non-Elimination Games**

**Description:** *Plan and implement inclusive games to ensure that students are provided with many opportunities for activity and skill development.*

***Example:*** *Continuous ball tag game in which tagging a player results in a point for the tagging team and the tagged player remains in the game vs ball tag game in which students sit on the side when they are tagged.*

| **Characteristics of  *Poor*** | ***Characteristics of  Moderate*** | **Characteristics of  *Excellent*** |
| --- | --- | --- |
| Students are eliminated and are sedentary for a period of time during an activity. | Some students are eliminated for a period of time during a limited number of activities during the lesson. | Students are never eliminated and sedentary during lesson activities. |

**Strategy 7: Modify Games to Maximise Activity and Skill Development**

**Description:** *Plan and implement game modifications (or adaptations) to ensure that students are active and are given many opportunities to practice the skill.*

***Example:*** *A cricket game in which there are multiple batters and wickets and the fielding team must constantly rotate positions vs a single batter and wicket, with stationary fielders.*

| **Characteristics of  *Poor*** | ***Characteristics of  Moderate*** | **Characteristics of  *Excellent*** |
| --- | --- | --- |
| Activities do not provide many opportunities for activity and skill development. | Some activities provide opportunities for activity and skill development. | All activities provide many opportunities for activity and skill development. |

**Strategy 8: Integrate Fitness into Activities**

**Description:** *Plan and implement fitness boosters to ensure that students are active and receive a fitness benefit when participating in activities.*

***Example:*** *Students complete fitness activities (e.g., hold a front support for 15 seconds) while waiting for their turn on the gymnastics apparatus vs students must stand in line waiting for their turn.*

| **Characteristics of  *Poor*** | ***Characteristics of  Moderate*** | **Characteristics of  *Excellent*** |
| --- | --- | --- |
| No modifications were made to replace sedentary waiting time with vigorous (high intensity) activity. | Some modifications were made to replace sedentary waiting time with vigorous (high intensity) activity, but further improvements in organisation could be made. | Modifications are made to activities to ensure that students increase vigorous (high intensity) activity and minimise sedentary waiting time. |

**Strategy 9: Choose Activities that Maximise Moderate-to-Vigorous Physical Activity**

**Description:** Choose sports within a broad category that are more active than others.

***Example:****Where possible choose soccer over American football,
Where possible choose golf over archery,
Where possible choose paddle tennis over volleyball.*

| **Characteristics of *Poor*** | ***Characteristics of Moderate*** | **Characteristics of *Excellent*** |
| --- | --- | --- |
| The nature of the sport/activity does not enable students to accumulate a reasonable level of physical activity, even in a well-designed lesson. | This sport/activity allows students to accumulate a reasonable level of physical activity in a well-designed lesson. | This sport/activity allows for students to accumulate a high level of physical activity in a well-designed lesson. |

**Principle 2: Building Competence**

**Strategy 1: Provide overview of units & lessons**

**Description:** Instructions ensure that students understand the activities that will take place across a unit or within a lesson.

***Example:****Unit: “This unit will be 6 lessons, we’ll spend the first 2 lessons…, then we’ll…, by the time we get to the last two lessons, you’ll be ready to…” Lesson: “Today we’re going to begin with…, then we’ll…, we’ll finish with…”*

| **Characteristics of  *Poor*** | ***Characteristics of  Moderate*** | **Characteristics of  *Excellent*** |
| --- | --- | --- |
| No overview is provided for the lesson. | An overview is provided, but might be confusing for some students. | A clear, concise overview of the lesson is provided. |
| An overview is provided, but is very confusing. | An overview is provided, but too much detail is included. |  |

**Strategy 2: Make behavioural expectations clear**

**Description:** When providing instructions, the teacher ensures that students understand what expected of them.

***Example:*** *“When you get into the sports court, there is a rack of basketballs. Start a game of 3 on 3 on each basket, but do move the cones that are in the middle of the court. We’ll use those in our second activity”.*

*“The boundaries of the court are marked by the white lines. I’ve also put orange cones around to make it even clearer****.”***

| **Characteristics of  *Poor*** | ***Characteristics of  Moderate*** | **Characteristics of  *Excellent*** |
| --- | --- | --- |
| Instructions to students are often overly complicated and difficult to understand. | Some instructions are clear, but others are confusing. | Clear instructions are provided for each activity. |
| Instructions often do not provide enough information for students. | Instructions contain only some of required information or some aspects are not as clear as they could be. |  |

**Strategy 3: Use questioning**

**Description:** The teacher asks questions that are designed to help guide students towards deeper understanding of a topic.

***Example:*** *“If you want to get some backspin on the ball, what would you do?”*

| **Characteristics of  *Poor*** | ***Characteristics of  Moderate*** | **Characteristics of  *Excellent*** |
| --- | --- | --- |
| Questioning is not employed. Instead answers are provided for students. | Questioning is employed, but only infrequently. | Open-ended questioning is employed and requires students to reflect on the topic in order to answer. |
| Questioning is employed, but answering requires little thought by the student. | Questioning is employed, but open-ended, thought provoking questions are mixed with closed questions or questions that require little thought by students. |  |

**Strategy 4: Provide effective positive feedback**

**Description:** Teachers provide specific, as opposed to vague, feedback that focuses on behavior (e.g., technique or effort) not ability, and is delivered in a private (one on one) setting when possible.

***Example:*** The teacher approaches a student as he is retrieving his basketball, “Great follow-through on that shot, Michael. You really extended your hand nice and high. Next time, try to jump even higher on the shot, so you can really release from the highest point possible. But, keep that great follow-through. That was really excellent.”

| **Characteristics of  *Poor*** | ***Characteristics of  Moderate*** | **Characteristics of  *Excellent*** |
| --- | --- | --- |
| Feedback is usually vague (e.g., “good job” and “nice one”). | Feedback is sometimes vague, but specific feedback is sometimes used. | Feedback is almost always specific. |
| Feedback usually focuses on students’ ability (e.g., “you’re a great shooter”). | Feedback sometimes focuses on students’ ability, but feedback on behavior is also provided at times. | Feedback almost always focuses on students’ behavior (e.g., “great effort there, Michael. You’re really improving the height in your jump shot”). |
| Feedback is delivered exclusively in a public setting where other students can hear. | Feedback occasionaly is delivered privately. | Feedback is regularly delivered privately. |

**Strategy 5: Provide effective corrective feedback**

**Description:** Positive feedback is provided before and after corrective feedback about technique. Feedback provides the student with specific information about how to improve.

***Example:*** “Nice extension on that serve, Amanda. Try to get that ball toss nice and high each time. Keep going, you’re really improving your serves.”

| **Characteristics of  *Poor*** | ***Characteristics of  Moderate*** | **Characteristics of  *Excellent*** |
| --- | --- | --- |
| Positive feedback is rarely or never provided alongside corrective feedback. | Positive feedback is sometimes sprovided alongside corrective feedback. | Positive feedback is regularly provided alongside corrective feedback. |
| Specific information about how to improve is rarely provided; only crticism of current behavior is provided. | Specific information about how to improve is sometimes provided. | Specific information about how to improve is regularly provided. |

**Strategy 6: Match task to ability level**

**Description:** The lesson offers opportunities for students of different skill levels to participate simultaneously in activities that are personally challenging, but realistically achievable (i.e., differentiation).

***Example:*** *If you feel like you’ve mastered this one with your dominant hand, try it with the other hand.*

| **Characteristics of  *Poor*** | ***Characteristics of  Moderate*** | **Characteristics of  *Excellent*** |
| --- | --- | --- |
| There is little opportunity for students of differing skill levels to attempt different tasks or different variations within a task. | Some opportunity is provided for students of differing skill levels to attempt different tasks or different variations within a task. | Most or all students have opportunities to participate in activities that personally challenging, but realistically achievable. |
| Little challenge is available to students with high skill levels or students with lower skill levels have little chance of success. |  | Many opportunities are provided for students of differing skill levels to attempt different tasks or different variations within a task. |

**Strategy 7: Limit peer comparison**

**Description:** The teacher avoids praising one student over others. The teacher limits public comparison of performance between students. The teacher avoids praise for ‘winning’.

**Example of poor practice:** “Who did more than 6? More than 10? More than 12? Wow. More than 20? Great job!” and “Ok, great job on the game of one on one. Winners move to their left to face their next opponent. Losers stay put.”

| **Characteristics of  *Poor*** | ***Characteristics of  Moderate*** | **Characteristics of  *Excellent*** |
| --- | --- | --- |
| The teacher often makes verbal peer copmparisons. | The teacher periodically makes verbal peer copmparisons. | The teacher never makes verbal peer copmparisons. |
| Activities are often structured in a manner that promotes peer comparisons. | Activities are sometimes structured in a manner that promotes peer comparisons. | Activities are rarely or never structured in a manner that promotes peer comparisons. |

**Strategy 8: Promote self-comparison**

**Description:** The teacher praises effort and improvement by an individual student. The teacher utilizes techniques that makes self-improvement clear to each student in a private manner**.**

**Example:** “Great effort there, Michael. You really went all out there”.

“OK, at the start of term, we did a series of fitness activities. Today we’re going to see how much you’ve improved on each task, so make sure you give it heaps!”

| **Characteristics of  *Poor*** | ***Characteristics of  Moderate*** | **Characteristics of  *Excellent*** |
| --- | --- | --- |
| The teacher rarely praises effort or improvement. | The teacher sometimes praises effort or improvement. | The teacher often praises effort or improvement. |
| The teacher rarely employs activities that allow students to gauge their improvement or the activities are structured in a way that make public comparisons likely. | The teacher rarely employs activities that allow students to gauge their improvement. | The teacher rarely employs activities that allow students to gauge their improvement. |

**Principle 3: Supporting Students**

**Strategy 1: Emphasise Fun and Variety**

**Description:** *Plan and implement activities which maximize fun, creativity, novelty, and variety. Where possible, start and finish the lesson with an enjoyable activity.*

***Example: 1) FUN*** *–“Okay guys, I’ve seen how most of you love playing bull-rush so today we are going to use it as our warm-up game”…. “To finish today’s lesson we’re going to have a game of modified dodge ball. I know it’s one many of you like.”*

***2) CREATIVITY*** *– “Today we are going to combine the skills we have been learning from AFL and Soccer into the one game. If the ball is on the ground, soccer rules apply (i.e. no hands). If the ball has been caught on the full I will blow my whistle indicating that AFL rules are now in play”.*

***3) NOVELTY and VARIETY****– “I know that we have been playing soccer for the past 3 weeks, but today we’re going to look at European Handball. I suspect it’s a game many of you haven’t played much before and it may help you develop new game awareness skills that you can use in games you already play, like soccer.*

| **Characteristics of  *Poor*** | ***Characteristics of  Moderate*** | **Characteristics of  *Excellent*** |
| --- | --- | --- |
| There appears to be little effort by the teacher to ensure student enjoyment (e.g., humour, popular games, interactive/tag games). | There is some effort by the teacher to ensure student enjoyment (e.g., humour, popular games, interactive/tag games), but some aspects are not well executed.. | The teacher employs strategies to ensure student enjoyment (e.g., humour, popular games, interactive/tag games), especially at the start and end of the lesson. |
| Lesson activities are standard with no use of creativity. | Creativity can be seen in some lesson activities. | Many lesson activities are creative and differ from regular games/activities students may partake in during their own time. |
| All lesson activities lack novelty and there is no evidence of variety. | Some lesson activities are novel and there is some evidence of variety, either within the lesson or from lesson to lesson. | Most lesson activities have novel aspects and there is substantial evidence of variety, either within the lesson or from lesson to lesson. |

**Strategy 2: Circulate Around the Class**

**Description:** *Ensure movement during PE lessons allowing observation and feedback to be provided equally to all students.*

***Example: 1)*** *Teacher walks amongst students and rotates his/her observation during lesson activities.*

| **Characteristics of  *Poor*** | ***Characteristics of  Moderate*** | **Characteristics of  *Excellent*** |
| --- | --- | --- |
| Teacher stands or sits in the one place throughout most of the lesson. The teacher does not circulate amongst students during lesson time | The teacher circulates and rotates observation amongst students some of the time. | The teacher circulates and rotates observation amongst students during most lesson activities. |
| The teacher interacts with a very limited number of students during the lesson. | The teacher interacts with some students during the lesson. | The teacher interacts with many students during the lesson. |

**Strategy 3: Provide Students with Opportunities to Make Choices**

**Description:** *Allow students to have a choice regarding unit topics and lesson activities. Also, provide students with options within the lesson (e.g., different tasks and/or and difficulty of tasks). Utilise free choice periods where students have freedom to choose and organise their tasks.*

***Example: 1)*** *“Okay everyone, today we are doing a fitness circuit. There are cards set-up around the court, on each card there is an activity with two levels of difficulty. Select the level of difficulty that you want when taking part in each activity.*

***2)*** *“Hi everyone, today is the last lesson of this term’s unit. As you will see, I have set up different pieces of equipment around the court. Today is going to be a free choice lesson. When I say go, you may all choose a piece of equipment and organise an activity you’d like to play.*

| **Characteristics of  *Poor*** | ***Characteristics of  Moderate*** | **Characteristics of  *Excellent*** |
| --- | --- | --- |
| There is little opportunity for students to make choices regarding lesson tasks or the level of difficulty in which they are performed. | Some opportunities are provided for students to choose lesson tasks or the level of difficulty in which they are performed. | Regular opportunities are provided for students to choose lesson tasks and the level of difficulty in which they are performed. |
|  |  | Students are provided with a period of free choice in which they can choose and organise their own lesson activities. |

**Strategy 4: Provide a Rationale and Emphasise Relevance.**

**Description:** *Instructions include a description of how and why lesson activities are important and relevant to students’ lives.*

***Example: 1)*** *“Now we are going to play a game of bull-rush. Not only will this activity warm up our muscles to help prevent injuries during today’s lesson, but it will also allow us to work on our footwork, speed, and agility, all which are valuable skills which can be applied to many different sports and games.*

*2) Oztag is a great game for all kinds of reasons. It’s great for your cardiorespiratory fitness and it’s really social – a game that you play after you leave school.*

| **Characteristics of  *Poor*** | ***Characteristics of  Moderate*** | **Characteristics of  *Excellent*** |
| --- | --- | --- |
| No rationale for lesson activities is provided to students and relevance to students’ lives is not explained. | Rationale is provided for some lesson activities with an occasional description of how and why activities are relevant to students. | A clear rationale is provided for all lesson activities and relevance to students’ lives is explained. |

**Strategy 5: Minimise Controlling Language and Behaviour**

**Description:** *When providing instructions and feedback to students, the teacher avoids language and behavior that students are likely to perceive as controlling.*

***Example:*  *1) “****Dave, I want you to lift your arm higher. If you don’t you’ll continue to miss the target”* ***VS*** *“Dave, if you’re looking to direct your shot more consistently, you could try lifting your arm higher.”*

***2)*** *“Everyone get over here on the baseline with me”* ***VS*** *“Everybody over to the baseline quickly please, we’ll get started as soon as possible.”*

| **Characteristics of  *Poor*** | ***Characteristics of  Moderate*** | **Characteristics of  *Excellent*** |
| --- | --- | --- |
| The teacher often uses controlling language when communicating with students. | The teacher sometimes uses controlling language when communicating with students. | The teacher rarely/never uses controlling language when communicating with students. |

**Strategy 6: Take the Students’ Perspective**

**Description:** *The teacher puts him/herself in the student’s position by empathising with their difficulties, engaging in conversations about their lives outside of PE, and managing their misbehavior respectfully.*

***Example: 1)*** *“Tim, I understand that soccer is not something you enjoy, but I have really seen your skills develop the past few weeks and the more you practice the better you will get”.*

***2)*** *“Hi Tim, I hear you’re going to be in the school play. That’s great. Are you excited about it?”*

***3)*** *“Tim, could you please come over here for a second… Tim, I am aware that you do not like netball that much, and I know you’d prefer to play something else. But, try to remember that other people do like the game and you misbehaving is disruptive for them.”*

| **Characteristics of  *Poor*** | ***Characteristics of  Moderate*** | **Characteristics of  *Excellent*** |
| --- | --- | --- |
| The teacher rarely recognises and shows empathy towards students’ difficulties. | The teacher sometimes recognises and shows empathy towards students’ difficulties. | The teacher often recognises and shows empathy towards students’ difficulties. |
| The teacher does not communicate with students about their lives outside of PE. | The teacher communicates with students about their lives outside of PE, but it is not clear to what extent there is genuine interest in the students’ well-being (e.g., few follow-up questions are asked). | The teacher communicates with students about their lives outside of PE and shows genuine interest in the students’ well-being. |
| The teacher shows little respect towards students when dealing with cases of misbehaviour. | The teacher sometimes manages students’ misbehaviour with respect, but in some instances this could be improved (e.g., handling in public vs private). | The teacher respectfully manages all cases of students’ misbehaviour in private away from other class members. |

**Principle 4: Reducing Transition Time**

**Strategy 1: Manage the Change Room Effectively**

**Description:** *Apply policies and practices that ensure students get changed into their PE uniforms efficiently.*

***Example: 1)*** *Allow students to arrive dressed in their PE uniform to eliminate the time lost when students are in the change room.*

***2)*** *“Okay everyone, as soon as you are dressed you can have free play until we start the lesson.*

| **Characteristics of *Poor*** | ***Characteristics of Moderate*** | **Characteristics of *Excellent*** |
| --- | --- | --- |
| The teacher does not provide behavioural expectations or monitor students’ behavior during changing procedures. More than 10 minutes of activity time is lost. | The teacher provides behavioural expectations but does not monitor students’ behavior during changing procedures. At least 5-10 minutes of activity time is lost. | The teacher provides behavioural expectations and monitors students’ behavior during changing procedures. Less than 5 minutes of activity time is lost. |
| School polices that slow down change room procedures have not been identified or addressed. |  | The school has identified and implemented policies that speed up change room procedures. |

**Strategy 2: Take the Roll While Students Are Active**

**Description:** *Plan and implement for students to be active as soon as possible (ASAP) at the beginning of the lesson and take the roll while they are active.*

***Example:*** *The* *teacher circulates amongst students marking the role while they are taking part in an active game of pac-man.*

| **Characteristics of *Poor*** | ***Characteristics of Moderate*** | **Characteristics of *Excellent*** |
| --- | --- | --- |
| All students are sedentary while the roll is marked. | Some students are sedentary while the roll is marked. | All able bodied students are active whilst the roll is marked. |

**Strategy 3: Early Activity Set-Up**

**Description:** *Where possible, set up equipment before the lesson begins. Also, set up equipment for the next activity before the previous one finishes. Involve non-participating and injured students in equipment set-up procedures to maximize efficiency.*

***Example: 1)*** *The teacher arrives at the sports court before the bell and sets up markers and equipment prior to the students arriving.*

***2)*** *The teacher begins setting up a grid for the next activity whilst the students are still active in the previous lesson component.*

| **Characteristics of  *Poor*** | ***Characteristics of  Moderate*** | **Characteristics of  *Excellent*** |
| --- | --- | --- |
| No equipment or space is set up prior to activities commencing and students must wait for the teacher. | Some activities have the required equipment and space set up before commencing. | All activities have the required equipment and space set up before commencing. |
|  |  | The teacher arrives at the lesson area and begins setting up space and equipment before the lesson has begun. |

**Strategy 4: Decrease Talk / Instructions**

**Description:** *Provide concise instructions and get activities started as quick as possible. At the beginning of the lesson, use warm-up games that require minimal instructions. Also, promote self-direction amongst students to reduce “teacher talk” time.*

***Example: 1)*** *“Okay guys, once you enter the gym, grab an activity card, choose an activity, and start as soon as you’re ready”.*

***2)*** *“Okay, we are now going to play a modified game of netball. The rules are: 1) No contact with the opposition; 2) Maximum of 2 steps with the ball; and 3) No passes to a player outside your third. We will discuss more rule as the game progresses. Once both teams are standing on their baseline we will begin.*

| **Characteristics of  *Poor*** | ***Characteristics of  Moderate*** | **Characteristics of  *Excellent*** |
| --- | --- | --- |
| Instructions are lengthy and over-complicated resulting in a large amount of student activity time lost due to teacher talk time. | Some instructions are concise, but others are over-complicated resulting in some student activity time lost due to teacher talk time. | Instructions are concise and kept to a minimum resulting in minimal student activity time being lost due to teacher talk time. |
| The teacher does not promote self-direction amongst students. |  | The teacher promotes self-direction amongst students. |

**Strategy 5: Form Groups Efficiently**

**Description:** *Plan and implement time efficient strategies when organising students into groups during PE lesson time.*

***Example:*** ***1)*** *“Okay guys, everybody form a group of 4, you have 5 seconds – 5….4….3…..etc. .”* Teacher sorts out any student who is not in a group*. “Okay, (injured non-participating students) Sandra, Charlie, and I will now come around and hand you a coloured tag. The colour you receive will be your team for the next activity”.*

***2)*** *“Okay everyone, we are now going to play a modified game of endball. You will be playing in the four teams that we organised earlier in today’s lesson”.*

| **Characteristics of  *Poor*** | ***Characteristics of  Moderate*** | **Characteristics of  *Excellent*** |
| --- | --- | --- |
| The teacher does not use strategies to promote the efficient formation of groups. | The teacher uses strategies to promote the efficient formation of groups during some team/group activities. | The teacher uses strategies to promote the efficient formation of groups during all team/group activities. |

**Strategy 6: Distribute Equipment Quickly**

**Description:** *Plan and implement strategies to assist with efficient distribution and collection of equipment during PE lesson time.*

***Example: 1)*** *“(injured/non-participating students) Charlie, Sandra, and I will now come around and hand each group a basketball”.*

***2)*** *“Before you get changed please place your basketball in one of the three ball bins up the front”.*

| **Characteristics of  *Poor*** | ***Characteristics of  Moderate*** | **Characteristics of  *Excellent*** |
| --- | --- | --- |
| The teacher does not implement strategies to assist with efficient distribution/collection of equipment. A considerable amount of student activity time is lost. | The teacher sometimes implements strategies to assist with efficient distribution/collection of equipment. Some student activity time is lost. | The teacher always implements strategies to assist with efficient distribution/collection of equipment. A minimal amount of student activity time is lost. |

**Strategy 7: Manage Water Breaks Efficiently**

**Description:** *Be conscious of the time lost and the behavioural problems that can arise when students stop for a water break. Provide clear instructions and guidelines to students to minimise time lost during water breaks.*

***Example: 1)*** *“Okay everyone, we are now going to take a quick 2 minute drinks break. I expect everyone to behave responsibly and follow the same rules we uphold during lesson time.”.*

***2) “****Nice work today. I just want to remind everyone to bring a water bottle to our next lesson. If we all have water bottles at our lesson area, our water breaks will be much shorter and we can stop for a quick drink more frequently”.*

| **Characteristics of  *Poor*** | ***Characteristics of  Moderate*** | **Characteristics of  *Excellent*** |
| --- | --- | --- |
| The teacher does not provide behavioural expectations or manage student behavior during water breaks resulting in a considerable loss of lesson activity time. | The teacher provides behavioural expectations but does not manage student behavior during water breaks resulting in some loss of lesson activity time. | The teacher provides behavioural expectations and manages student behavior during water breaks resulting in minimal loss of lesson activity time. |
| Few students bring a drink bottle to class / The teacher does not encourage students to bring a drink bottle to their lesson area. | Some students bring a drink bottle to class / The teacher encourages students to bring a drink bottle to their lesson area. | Most students bring a drink bottle to class. |
